# Loneliness and Social Support among the Middle-Aged and Elderly People with Visual Impairment

**DOI:** 10.3390/ijerph192114600

**Published:** 2022-11-07

**Authors:** Hui-Ying Chu, Hui-Shan Chan

**Affiliations:** 1 Department of Living Services Industry, Tainan University of Technology, Tainan City 710, Taiwan; 2 Department of Applied Cosmetology, National Tainan Junior College of Nursing, Tainan City 700, Taiwan; 3 Department of Special Education, National Tainan University, Tainan City 710, Taiwan

**Keywords:** middle-aged and elderly people with visual impairment, loneliness, social support

## Abstract

Loneliness is associated with depression, sleep disturbance, and an increased risk of cardiovascular disease, and it is a global public health problem. Since physical and mental health have a great impact on loneliness, middle-aged and elderly people who are blind or visually impaired may be more affected by loneliness. Previous research has confirmed that effective social support can enhance physical and mental health and alleviate the negative effects of life stress. Therefore, in this study, we applied a cross-sectional design where data were collected using questionnaires completed in person, by phone, or online for a total of 456 middle-aged and elderly people with visual impairment. We found that the enrolled participants who were unemployed, lacked a stable source of income, lived alone, or were unable to move independently were prone to experiencing high levels of loneliness and low social support, which highlights the necessity of interventions such as counseling to alleviate the sense of loneliness in such groups. During the COVID-19 pandemic, social support measures to reduce the sense of loneliness should be highly encouraged to ensure that middle-aged and elderly people with visual impairment can continue to live independently, and social support seems to be an important factor.

## 1. Introduction

According to the World Health Organization (WHO), at least 2.2 billion people globally have visual impairment caused by nearsightedness or farsightedness. In at least 1 billion of these cases, visual impairment could have been prevented, or has yet to be addressed [1,2]. The majority of people with visual impairment and blindness are over the age of 50 years [1,2,3], and population growth and aging are expected to increase the number of people developing visual impairment. The weighted number of Chinese adults aged 18 years and older with visual impairment has been estimated to be 16.9 million, one third of whom are blind. In addition, the prevalence rates of low vision and blindness in adults aged 40 years and older have been reported to be 4.9–5.3% and 1.0–1.9%, respectively [4,5,6]. In the United States, the Estimates of Older People with Vision Loss from the American Community Survey (ACS) showed that 3.2 million people (6%) aged 65 years or older experienced vision loss in 2019, and the prevalence of visual impairment increasing with age [7,8]. According to data from the Ministry of Health and Welfare of Taiwan in 2021, the number of people with visual impairment increased from 38,747 in 2000 to 55,462 in 2021, of whom 64.5% and 79.8% were over 50 years of age, respectively [9].

Visual impairment severely impacts the quality of life of adults, as adults with vision impairment often have lower rates of workforce participation and productivity and higher rates of depression and anxiety. In the case of older adults, visual impairment can contribute to social isolation, difficulty walking, a higher risk of falls and fractures, and a greater likelihood of early entry into nursing or care homes [2,3]. These findings illustrate that the advent of an aging society has led to a rise in the number of people with visual impairment, and this issue should be given greater attention.

Loneliness refers to feelings of depression, melancholy, low spirits, and emptiness [10], which encompasses pessimism, separation, and isolation, and is often a state of unmet personal or social emotional needs [11,12]. Blazer [13] reported that loneliness is an important factor influencing depression, and that it has a significant impact on human physical and psychological health. Blazer further stated that people who feel lonely have significantly higher rates of cardiovascular diseases, hypertension, depression, and mortality than those with intimate relationships [13,14,15]. Studies have suggested that people with visual impairment may be at a greater risk of loneliness, as they may have fewer opportunities for learning and building social skills than the average person of their age [16]. Brunes et al. [17] found that 1/6 of visually impaired adults suffered from moderate or severe loneliness, suggesting that the prevalence of loneliness was consistently higher in people with visual impairment than in the general population of all ages [18].

Among the various theories regarding the causes of loneliness [18,19,20,21], the cognitive discrepancy approach emphasizes that the discrepancy between personal cognitive and ideal interpersonal relationships is one of the causes of loneliness [22]. Moreover, loneliness arises from the negative effects of interpersonal interactions due to the discrepancy between cognition and ideal [14]. In contrast, the social needs approach of Weiss [22] stated that individuals satisfy their needs in social relationships through social provisions with different functions, including attachment, social integration, nurturance, reassurance of worth, and reliable alliance and guidance. Loneliness is caused by a lack of social provisions, which emphasizes the lack of social relationships. Loneliness consists of emotional isolation/emotional loneliness and social isolation/social loneliness. The former refers to the lack of close emotional attachments, causing anxiety and inner emptiness, and only when new intimate attachments are created or relationships are restored with the original attachments can emotional isolation be alleviated [21,22]. The latter is caused by a lack of social networks, the inability to feel connected to others, and the tendency to become bored, marginalized, and isolated [21].

People who are visually impaired are more likely to feel lonely and may have fewer opportunities to learn and socialize than sighted people [23]. The risk factors associated with visual impairment, such as poor health, low financial status [24], and unfavorable relationship building [25], are also strongly associated with the feelings of loneliness [26]. The risk of loneliness is especially high in people between the ages of 36 and 50, those who have been bullied, physically or sexually abused, and who are blind or have other impairments [24]. These findings highlight the psychological and physical impacts of loneliness on people with visual impairment, and that greater attention and prevention are required.

Previous studies have shown that social support can enhance physical and mental health and mitigate the negative effects of life stress [27,28,29,30]. The buffer theory of House [31] suggests that for an interpersonal transaction, social support should include both direct effect and buffering effect models. The former refers to the direct effect of social support on reducing stress and enhancing physical and mental health by satisfying the need for safety, recognition, and a sense of belonging; while in the latter, social support plays the role of a buffer, which has an indirect positive effect on physical and mental health and life adjustment when individuals encounter stressful situations. Moreover, psychological support (love and understanding) has a protective effect on well-being, thus preventing feelings of loneliness. Receiving help when needed not only develops self-esteem and self-confidence, but also increases the sense of security and control over oneself and the environment [32]; the higher the level of social support for older adults, the higher their life satisfaction. The friendship, help, and relief that older adults receive from genuine interpersonal interactions can promote their psychological well-being [33]. In addition, adequate social support is associated with successful aging, and support from friends has been shown to be more important than support from family members, while inadequate social support may impair physical functioning [34].

The importance of the association between loneliness and social support in young people with visual impairment was reported in a longitudinal study, which found that perceived parental support decreased linearly from adolescence to young adulthood, while peer-friend support increased. Moreover, in middle-aged and older people, the support of similarly aged friends was a better indicator of the risk of loneliness in later life than parental support, emphasizing the importance of establishing and maintaining lifelong peer relationships [16]. Other studies have confirmed that social support is associated with good health, including lower mortality and faster recovery [35,36,37], which suggests that with adequate social support, people have a more optimistic outlook on their physical health. These findings highlight the various forms of support and assistance that individuals can receive from different interpersonal interactions, in addition to providing stress-buffering benefits regarding recovery from injury. In recent years, increasing attention has been paid to the protection of the rights and interests of disadvantaged groups and special communities worldwide; however, fewer studies have investigated issues related to social support and loneliness in the visually impaired. Therefore, in this study, we enrolled middle-aged and elderly people with visual impairment in Taiwan to examine the correlations among demographics, loneliness, and social support. With the aging society, it is expected that greater attention will be paid to the problem of loneliness and related factors in older people with visual impairment in order to improve their physical and mental health.

## 2. Research Materials and Methods

### 2.1. Design

This study was approved by the Human Experiment and Ethics Committee of National Cheng Kung University, applied a cross-sectional design, and was conducted between September 2021 and April 2022. A questionnaire survey was adopted for data collection via questionnaires completed in person, by phone, or online. A total of 502 middle-aged and elderly people with visual impairment were recruited from various institutions in Taiwan, and 456 valid questionnaires were collected after the incomplete responses were removed.

### 2.2. Recruitment

This study conducted non-random purposive sampling at the Institute of the Blind of Taiwan and the Mu-Kuang Rehabilitation Center for the Blind, which are organizations for the visually impaired, and the Visual Impairment Massage Professional Union of various counties and cities. The study participants were selected based on the following criteria: (1) the participants were visually impaired persons with a government issued Disability Card, and the age limit was 45–70 years old; (2) the study sample was limited to persons with a high level of impairment and those with multiple disabilities or persons under 20 years of age with a Disability Card were excluded; (3) the participants were able to communicate and express their feelings naturally in Mandarin or Taiwanese. The sample size of this study was estimated using the G-power analysis program. The effect size was calculated as 0.136 by R2 based on a pretest sample of 60 participants. This study also referred to Cohen [38] to set the Cronbach’s α at 0.05 and 1-β at 0.95 [38] and estimated the total sample size to be 382.

### 2.3. Measures

The research instruments included basic personal information, “The UCLA Loneliness Scale Version 3”, as developed by Russell [39], and the “Interpersonal Support Evaluation List” (ISEL), as proposed by Cohen et al. [40]. The details are described as follows:

#### 2.3.1. Basic Personal Information

Gender, age, educational level, marital status, job types, monthly salary, religion, lifestyle, ability to move independently, the cause of blindness, and important support and companionship in life.

#### 2.3.2. The UCLA Loneliness Scale Version 3

The UCLA Loneliness Scale Version 3 developed by Russell [39] was adopted as the measurement instrument of this study, as it is one of the most widely used measurement instruments in current research, with good reliability and validity for different research groups. The UCLA Loneliness Scale Version 3 was translated into Chinese in 1999 by Taiwanese researchers, Su-Hong Chang and Mei-Sang Yang [41], with permission from Russell. The scale was developed to measure the degree of loneliness and consists of 20 questions, which are rated according to the subjective feelings of the individual, as “never”, “rarely”, “often “, and “always”, and scored 1, 2, 3, and 4, respectively. The higher the scale score, the higher the degree of loneliness. According to Perry’s [42] loneliness classification scheme, a score of 20–34 indicates a low level of loneliness, 35–49 indicates a moderate level, 50–64 indicates a moderately high level, and 65–80 indicates a high level.

The validity and reliability of this scale were tested by expert validity and Cronbach’s α coefficients, and its validity and appropriateness were assessed by two caregivers, one social worker, and three experts in teaching the visually impaired. The content validity index (CVI) value was 0.83, and the overall Cronbach’s α reliability was 0.85, indicating that the scale had good stability. The t-values in the item analysis were 3.92–11.76 (*p* < 0.05), indicating good discrimination. This questionnaire was tested for reliability and validity, and then developed into a formal questionnaire.

#### 2.3.3. The Social Support Scale

This study was originated from the Interpersonal Support Evaluation List (ISEL) [41], as developed by Cohen et al. [38], which is a multi-dimensional scale that measures the individual’s perceived availability of social resources and support in the face of stressful events. The original scale contains 40 items and is divided into four subscales, which measure emotional or appraisal support, instrumental or tangible support, companionship or belonging support, and self-esteem maintenance. The Chinese version was later translated and revised by Chen, Tseng, Wang, and Lee [43], and contains the same four subscales as the original scale, with a total of 16 questions. This version has the same pattern of questions as the original scale, and both use a four-point Likert scale ranging from strongly agree (4 points) to strongly disagree (1 point). There are 9 reverse narrative questions. The total score of the scale ranges from 16 to 64, with higher scores indicating higher levels of perceived social support, and higher scores for the sum of all subscales indicating higher levels of overall social support.

ISEL is divided into: (1) emotional or appraisal support, which assesses the degree of emotional support available to individuals in times of need; (2) instrumental or tangible support, which is intended to understand the extent to which financial, tangible, and resource support is available in practical situations; (3) companionship or belonging support, which is intended to understand the extent to which individuals feel they belong to a group and are accompanied; and (4) self-esteem support, which is intended to understand the extent to which an individual’s self-esteem is maintained when spending time with relatives and friends.

Cohen et al. [40] conducted general population testing and constructed the validity and reliability data for this scale. The calculated alpha value of internal consistency was 0.88–0.90 for the total scale, 0.70–0.80 for the subscales, and the correlations among the four subscales were 0.30–0.50, indicating good independence among the subscales.

This study used ISEL, as proposed by Cohen et al. [40], which was translated and revised into a Chinese version with a 0.92 correlation with the original scale. Chen et al. [43] used postpartum depressed women as a pilot study with a retest reliability of 0.77 and an internal consistency alpha value of 0.81. In this study, the formal administration of the questionnaire was also analyzed by “expert validity” testing, and the CVI value was 0.87, the t-value in the item analysis was 4.30–18.63 (*p* < 0.05), and the Cronbach’s α value was 0.88, which showed good discrimination. The questionnaire was tested for reliability and validity, and then developed into a formal questionnaire.

### 2.4. Statistical Analyses

This study used IBM’s SPSS, Version 20.0 (SPSS Inc., Chicago, IL, USA) for data entry and analysis, and the main statistical methods were as follows: The percentage was used to describe the distribution of the demographic variables; the average mean and standard deviation were used to describe the scores of the scales; one-way ANOVA was conducted to compare the differences between the scores of the groups, and whether the differences between the groups reached a significant level; and Scheff’s post hoc comparison was applied. The interdependence between two or more variables was analyzed by Pearson’s product difference correlation, and stepwise multiple regression analysis was used to understand the causal relationship between the predictor variables within the regression model, as well as their explanatory predictions.

## 3. Results

### 3.1. Analysis of Participant Demographics

Table 1 provides the information for the 456 middle-aged and elderly participants with visual impairment, where 59.6% (*n* = 272) were male and 40.4% (*n* = 184) were female; the average age was 54.1 years; 52.6% (*n* = 240) were married; the majority (67.4%) of the participants (*n* = 307) were religious, where the largest number of participants (*n* = 128, accounting for 28.1%) believed in Taoism; 42.9% (*n* = 196) of the participants had graduated from senior high school and most (55.7%) of the participants were blue-collar workers (*n* = 254). The average monthly salary of the participants was USD 737.2; 76.8% were living with family members; parents and spouses were their most important support and companions in life, followed by children, siblings, relatives and friends, and volunteers. Among them, 33.8% of the visually impaired were not able to act independently, 66.2% were able to act independently, most of the visually impaired used a cane as an aid to go out; 14.6% used their residual vision, and a smaller number used a guide dog. The causes of severe visual impairment or blindness were diseases, with the highest percentage of 54.8%, followed by congenital blindness at 33.8% and accidents at 11.4%, including car accidents, fires, high temperatures, occupational injuries, crashes, falls, and other incidents.

### 3.2. Analysis of the Differences in Loneliness Scores and the Loneliness Degree of the Participants according to Different Sociodemographic Characteristics

As show in Table 2, among the participants, 19.7% (*n* = 90) reported a low level of loneliness, 49.1% (*n* = 224) a moderate level of loneliness, 29.4% (*n* = 134) a moderately high level of loneliness, and 1.8% (*n* = 8) reported a high level of loneliness.

The overall mean loneliness score was Mean = 44.05, with the loneliness scores ranging from 22 to 70 (median = 45), indicating that the participants experienced loneliness at moderate to high levels.

As shown in Table 3, there was a difference in loneliness scores between salary, religion, lifestyle, and ability to move independently (*p* < 0.001), and data analysis showed that those with no regular job and low monthly salary had higher loneliness; those who lived alone, with no ability to move independently and no religion had higher loneliness. There were no significant differences in loneliness with gender, age, educational level, marital status, types of job, religion, or cause of visual impairment (*p* > 0.05).

A monthly salary of USD 525 and below denotes a low salary, USD 526–1050 denotes a moderately low salary, USD 1051~1580 denotes a moderate salary, and USD 1580~2100 (and above) denotes a high salary.

### 3.3. Analysis of Variance in Social Support Scores

As shown in Table 4, the overall mean score of social support for the visually impaired was 38.63 (SD = 9.95), ranging from 16 to 63 (median = 38.63), indicating that the social support of the visually impaired was at a low-to-moderate level. The scores for each of the four dimensions of emotional or appraisal support, instrumental or tangible support, companionship or belonging support, and self-esteem support were 9.56 (SD = 2.89), 9.20 (SD = 3.52), 9.74 (SD = 3.54), and 9.96 (SD = 2.94), respectively, with the scores ranging between 4–16, 4–18, 4–39, and 4–16. The mean scores for self-esteem were higher (9.96 ± 2.94), while the mean scores for instrumental or tangible support were lower (9.20 ± 3.52).

As shown in Table 5, the social support scale differed by educational level, marital status, having a job or not, salary income, lifestyle, and ability to move independently (*p* < 0.001); data analysis showed that those with lower socioeconomic status, no fixed income, living alone, and lack of ability to move independently had lower social support scores. There was no significant difference between social support and gender, age, types of job, religion, or cause of visual impairment (*p* > 0.05).

### 3.4. Analysis of the Relationship between Loneliness and Social Support

As shown in Table 6, there was significant negative correlation between loneliness and social support (rs = −0.572, *p* < 0.001), where loneliness was negatively correlated with emotional or appraisal support (rs = −0.471, *p* < 0.001), instrumental or tangible support (rs = −0.556, *p* < 0.001), companionship or belonging support (rs = −0.521, *p* < 0.001), and self-esteem (rs = −0.608, *p* < 0.001). This indicates that the less social support the visually impaired persons felt, the higher their feeling of loneliness.

### 3.5. Demographic Interaction Analysis

**Figure 1:**
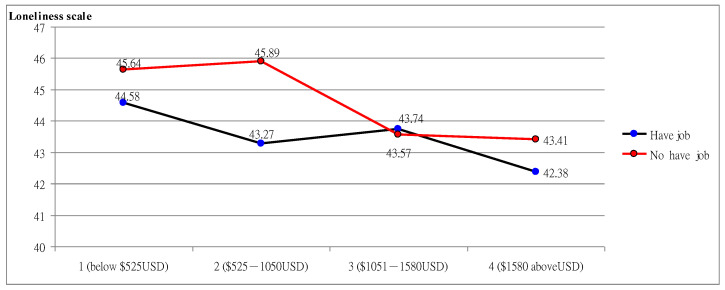
The interaction of work and income on loneliness.

As shown in Figure 1, demographic interaction analysis found a significant difference in loneliness scores. The effect of “having a job or not” was significant F = 8.211 *** (*p* = 0.000); the effect of monthly salary F = 5.09 ** (*p* = 0.001) was significant; and the interaction effect of “having a job or not” and “monthly salary” was significant F = 3.25 * (*p* = 0.030). The overall trend varied with monthly salary. The scores of those “having a job with a high monthly salary” decreased from 44.58 to 42.38 with lower loneliness, while the scores of those “not having a job with low monthly salary” decreased from 45.64 to 43.41 with higher loneliness, which validated that loneliness was affected by the interaction between “having a job or not” and “monthly salary”.

**Figure 2:**
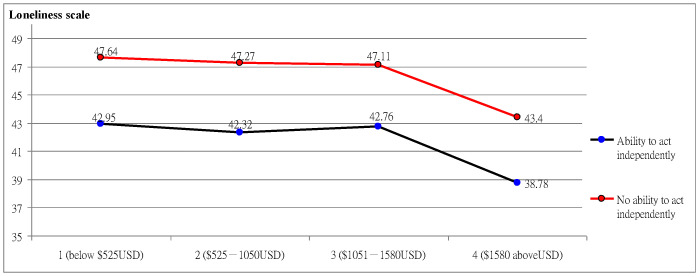
The interaction between the ability to move independently and the monthly salary on loneliness.

As shown in Figure 2, the interaction analysis of demographic variables found a significant difference in loneliness scores. The effect of “ability to move independently” was significant F = 16.96 *** (*p* = 0.000); the effect of monthly salary was significant F = 6.29 ** (*p* = 0.001); the interaction effect of “ability to move independently” and “monthly salary” was significant F = 5.25 * (*p* = 0.011), and the overall trend varied with monthly salary. The scores of “those who had the ability to move independently with a job and high monthly salary” decreased from 42.95 to 38.78, while the scores of “those who did not have the ability to move independently with a low monthly salary” decreased from 47.64 to 43.40, and their loneliness was higher, which verified that loneliness was affected by the interaction between “ability to move independently” and “monthly salary”.

### 3.6. Regression Analysis of Social Support on Loneliness

As shown in Table 7, a multiple stepwise regression analysis was conducted to understand the predictive power of each dimension of social support on loneliness. The results showed that three of the four predictor variables were effective in predicting loneliness after they were entered into the regression equation, namely, self-esteem (F = 40.118, *p* < 0.001), instrumental or tangible support (F = 30.326, *p* < 0.001), and companionship or belonging support (F = 26.385, *p* < 0.001). The multivariate correlation coefficient R was 0.457 and the coefficient of determination R square was 0.198, indicating that the loneliness amount of variance explained was 19.8%. In terms of the explanatory amount of individual dimensions, “self-esteem” had the best predictive power with an explanatory power of 11.7%, while the remaining were “instrumental or tangible support”, and “companionship or belonging support” with an explanatory power of 5.00% and 4.0%, respectively. Further analysis showed that the standardized Beta coefficients were all negative, indicating that the predictor variables had a negative influence on the effector variables, i.e., the higher the level of perceived self-esteem, instrumental or tangible support, and companionship or belonging support, the lower the level of loneliness, and “self-esteem” had the highest negative predictive power.

## 4. Discussion

This study examined the loneliness and social support of middle-aged and elderly people with visual impairment in Taiwan. The research sites included the Institute of the Blind of Taiwan, the Rehabilitation Center for the Blind, organizations for the visually impaired, and the Visual Impairment Massage Professional Union of various counties and cities. The sample characteristics were similar to those of other studies of the visually impaired, meaning middle-aged and elderly people with severe visual impairment or blindness. Nearly half of the participants had a low economic level, more than one-third were currently unemployed, and the majority of those who were employed were engaged in massage. As most vocational training and employment opportunities for the visually impaired in Taiwan are in massage occupations [44,45,46], in addition to the influence of government policies, some people with visual impairment think that it is easier to learn or have better income by engaging in massage nurturing education, or they do not develop other specialties themselves, thus, they choose to engage in massage [46,47]. The low employment rate of physically and mentally handicapped people has been a common phenomenon in many countries [48,49], and the same is true for people with visual impairment [49,50,51]. In addition to individual physiological factors, social factors cannot be ignored as the reasons for the low employment rate of people with visual impairment and their inability to integrate into a diverse workplace.

The research results of loneliness show that the participants had moderate and moderately high levels of loneliness, and the percentages were 49.1% and 31.2%, respectively. These results are higher compared to the data of Brunes [17], who mentioned that the prevalence rates of moderate and severe loneliness in the visually impaired population were 28.7% and 19.7%, respectively [17,24]. The visually impaired population in this study seemed to have a higher level of loneliness. Heppe et al. [16] showed that visually impaired youth were more likely to experience loneliness in their lives, as compared to peers. In particular, the prevalence of loneliness was as high as 54% in visually impaired people over 55 years of age. People with visual impairment were less resilient to loneliness, received less social support, and experienced more depression than non-lonely older people [52], suggesting a higher prevalence of loneliness in visually impaired older adults.

The cognitive discrepancy approach theory mentions that loneliness is a discrepancy between personal cognitive and ideal interpersonal relationships [22,53]. The degree of discrepancy depends on personal perceptual judgments [20] and is mainly a painful negative experience of separation between the mind and other people or things, including loneliness, emptiness, depression, or low spirits [54,55,56]. This situation is more pronounced in visually impaired older adults, as people with severe visual impairment are more likely to feel lonely because they cannot clearly see each other’s facial reactions. People with blindness or low vision may have fewer opportunities for learning and socialization than the average sighted person [23], and the risk of unemployment or fewer employment opportunities associated with disability is among the factors that are strongly associated with feelings of loneliness [57]. In this study, the degree of loneliness was correlated with job occupation, monthly salary, religious beliefs, lifestyle, and the ability to move independently, and the results show that people who were unemployed, lacked stable economic resources, lived alone, or were unable to act independently were more likely to have high loneliness. People with visual impairment are physically disadvantaged, as compared to the general population, and it is not easy for them to find jobs suitable for themselves. Moreover, there are some factors in the workplace that lead to employment bottlenecks and problems, meaning barriers that are difficult to overcome for people with various impairments [58,59]. The visually impaired face high unemployment rates and difficulties in finding a job [59].

People with visual impairment who live alone have a high level of loneliness, which is presumably due to a lack of adequate social networks or a sense of belonging to a group. In addition, those who lack the ability to move independently are less likely to engage in leisure activities because they are less mobile, or do not have sufficient financial resources to meet their social needs, and thus are more likely to feel isolated by their environment when they perceive their social connections as weak. Weiss [21] suggested that people who do not have a satisfactory social network tend to feel bored, purposeless, or even isolated and marginalized, and it has also been suggested that people who live alone are more dissatisfied with their relationships than those who live with others [60], which increases their feelings of loneliness.

This study found that religious people had low loneliness, which is the same as the results of other studies where people received more community resources and social connections through religious activities [61]. In particular, religious involvement may be a protective factor against poor physical and mental health in the elderly population, and those with high religious involvement are less likely to suffer from depression and have fewer physical health problems [62,63,64,65]. The low level of loneliness among the religious people with visual impairment in this study suggested a supportive strength from religious beliefs. In addition, regression analysis by Brennan [66] found that religiosity had a buffering effect on negative life experiences, socio-demographic variables, life stress, religious belief, and social support among middle-aged and elderly people with visual impairment, which is similar in the results in this study.

Social support has the ability to buffer individuals in stressful situations and reduce subsequent emotional reactions and coping behaviors [67]. This study attempted to extend social support to middle-aged and elderly people with visual impairment, which is a topic that has received little attention to date. The overall social support scores of the participants in this study were lower than the normal level of the average adult [68], suggesting that middle-aged and elderly people with visual impairment have a relatively low level of social support. This is in line with the research results of Verstraten et al. [52], who mentioned that those with less social support tend to feel more loneliness. In addition, compared to their sighted peers, visually impaired older adults are less likely to engage in too many social activities, have less contact with friends, and have smaller friendship networks [52,68,69,70]. The lower level of subjective perceived social support among middle-aged and elderly people with visual impairment may be due to the life situations they face. Wortman and Conway [71] noted that subjects who had experienced recovery from physical illness had a strong sense of need for social support, but they perceived difficulty in meeting this need. From the standpoint of the companion or caregiver, the possibility of being blamed for not putting in enough effort can contribute to the fear of providing social support. People with severe illness or poor resilience often have a strong desire for social support and are the most neglected groups [71]. In the same way, people with severe visual impairment face a loss of visual function and have an expectation of social support, but they perceive difficulty meeting this need, which may come from the hesitation of others, or the dislike of the disability. In addition, the COVID-19 pandemic has changed lifestyles by requiring social distance and more isolation, resulting in the stagnation of some jobs, a sharp decrease in economic income, and other crises. The stress and impact of life can be understood in the same way.

The levels of social support in this study were in the following order: self-esteem > companionship support > emotional support > instrumental support, which indicated that the participants’ subjective perceptions of self-esteem were slightly higher than the overall social support. However, the statistical data showed that the differences between the means of the first four types of support were small and not statistically significant. The content of the questionnaire items revealed that the participants’ life with friends and relatives, their levels of personal satisfaction, and self-esteem maintenance were influenced by various factors, such as support from friends of the same age [72] and their ability to move independently in daily life [73,74]. Question 8 of the questionnaire, “I am more satisfied with my life than most people”, had the lowest score because the loss of vision makes simple daily tasks seem difficult or impossible to perform, which leads to low life satisfaction [74], meaning they may experience a disproportionate number of negative interactions compared to their sighted peers, and therefore exhibit lower self-esteem [75]. Question 16, “I can’t keep up with my friends” indicated that dependence on others may lead to lower self-esteem in people with visual impairment, especially if they need help to complete their daily tasks [75].

Companionship support refers to the degree of companionship that an individual feels that he or she belongs to a group and is accompanied. The companionship of friends can reduce feelings of loneliness in middle-aged and elderly people with visual impairment living alone, and this finding is consistent with that of foreign studies [52]. This result shows that the most direct means of social participation, such as with workplace colleagues, interactive neighbors, or friends and volunteers at religious sites, can enrich the lives of middle-aged and elderly people with visual impairment.

In terms of emotional support, family members provided most of the emotional support in this study. From learning about the fact of blindness, the visually impaired went through psychological adjustments to gain re-adaptation, family companionship, and religious beliefs. Family members, significant others, and growth groups played an important role in social support [76]. The most important support and companions in the participants’ lives were their spouses and parents, followed by children, siblings, relatives and friends, and volunteers. Married people received most of their family support from their spouse and children, while those who lived alone or were widowed received family support from parents, siblings, relatives, friends, and volunteers, indicating the principle of supportive substitution when close family members, such as spouse and children, were not available [77], or more distant family members or non-family members were used as support resources. In the current social model in Taiwan, where adult children leave home to work in urban areas and children are unable to provide real-time care for their parents, siblings are closer to the visually impaired elderly than children and are more likely to share the values and attitudes of the middle and older aged groups, meaning age-related issues are more easily understood.

Instrumental support includes financial support, physical support, and resources. Financial problems and placements were often burdensome to the family role of the visually impaired and were often a source of other family problems [78,79,80]. Literature on the visually impaired suggests that the residual vision of the visually impaired affected their employment. Employers were generally less inclined to hire the totally blind, and some employers equated the ability to work with the residual vision of the blind [81,82,83]. Consequently, employment difficulties affected income and life support. Question 3, “If I get sick, it is hard to find someone to take me to the hospital,” and Question 7, “If I get sick, there is no one to help me with my daily chores,” illustrated the urgent need for the principle of supportive substitutions for people with visual impairment who live alone and have no spouse or children [77,84], and siblings, relatives, friends, and volunteers can work as a source of support and assistance.

In terms of demographic characteristics, the higher-educated group had more social support than the other groups due to the integrated educational approach of colleges offering people with visual impairment more opportunities to interact with resources from the school and the social system [85]. Participants who were married, able to work, and able to act independently received more social support, which may be related to better socioeconomic status and interpersonal skills. Those with lower socioeconomic status, no regular income, living alone, and lacking the ability to move independently received lower social support, which is similar to the study of Gong, Ni, and Wu [86], where a strong relationship was found between sociodemographic characteristics and visual impairment. This study’s finding of a lower level of social support among middle-aged and older participants is consistent with other studies [87,88], reflecting the fact that middle-aged and elderly people with visual impairment have less social contact and social interaction as they age, which is the reason for the decrease in social support.

Regarding the relationship between loneliness and social support, a negative correlation between loneliness and overall social support was found in this study, confirming that social support can play an important role in the loneliness of middle-aged and elderly people with visual impairment. Visual impairment may lead to the feelings of self-abasement, low self-esteem, or depression [89]. The prevalence of loneliness among visually impaired individuals over 55 years of age was as high as 54%, and they received less social support and had more depression than non-lonely visually impaired older adults [52]. Thus, it is recommended to help older visually impaired individuals with low socioeconomic status and a lack of ability to move independently for them to feel a sense of self-esteem, and to provide financial support, medical assistance, living assistance, or other tangible support, as well as more emotional support by making the most of existing resources.

The visually impaired are relatively disadvantaged in terms of physical conditions, and they have the more difficult barriers to overcome among all types of physical and mental disabilities [58]. As they are less likely to be hired by general businesses [90,91] it is not easy for them to find suitable jobs, and most middle-aged and elderly people with visual impairment in Taiwan work in massage services. During the COVID-19 pandemic, some of the work has been halted due to isolation requirements, resulting in a decrease in economic income, which is also a crisis and adversity caused by the pressure of life.

Taiwanese society should focus on improving the policies related to community development and family support to enhance the care services for elderly with visual impairment. Volunteers or friends, neighbors, and social workers should be recruited to visit the elderly with visual impairment on a regular basis to provide the assistance they need. The elderly with visual impairment are also encouraged to participate in community recreational activities. In addition, this study was conducted during the COVID-19 pandemic; thus, while the isolation and social distancing practices during the pandemic may have reduced the spread of the virus, it created a sense of social disconnection and loneliness [92]. There is also an Asian cultural emphasis on harmony within social groups, which means that when these visually impaired older adults encounter difficulties, they may rarely seek help or confide in others. Therefore, giving visually impaired older adults more social support and teaching them to use resources effectively in their lives may be an effective strategy to reduce their loneliness.

### Study Strengths and Limitations

The advantages of this study are the large sample size, the use of verified effective tools, and the provision of relevant information and comments from several Visual Impairment care team members, effectively estimating the sense of loneliness in the middle-aged and elderly Visual Impairment groups. However, there are still several research limitations: First, compared with ordinary adults, patients with severe visual impairment belong to the category of severe disabilities (hearing disabilities, intellectual disabilities, and physical disabilities). The samples were scattered. This study had to enroll the respondents through Rehabilitation Institutes for the Blind and Visually Impaired Organizations. Thus, the findings may not be generalizable to visual impaired who live in other regions of Taiwan. Second, there is no clear connection between loneliness and the visual impairment. However, when people with visual impairment lack a job income, live alone or lack care, are unable to act independently or are alienated from others, and lack social support, they are likely to experience a higher sense of loneliness. We applied a cross-sectional study that is unable to determine the cause-and-effect relationship, although some confounding factors were controlled so the analyses might be affected by the residual confounding factors. Third, the subsequent studies may include the identification of the health issues to enhance the research integrity.

## 5. Conclusions

This study found that middle-aged and elderly people with visual impairment tend to feel lonely and their social support level is low. Increasing social and family support may be an effective strategy to reduce loneliness. Loneliness and social support differed across the visual impairment with different sociodemographic characteristics such as unemployment, lacking stable economic resources, living alone, or being incapable of acting independently making people prone to become the group with high loneliness and low social support. This highlights the need for giving priority to counseling such groups or providing necessary interventions to reduce their loneliness.

Those with religious beliefs had a lower sense of loneliness, while those who were able to move independently and engage in more leisure or social activities felt more socially supported. The elderly with visual impairment who lacked financial stability were less able to afford living expenses and participate in social activities, making them more likely to feel marginalized and isolated in their environment when they perceived a weaker connection to society. Family members, parents, spouses and children provide most of the emotional support, and it means that family members’ companionship and support can help the visually impaired to have a positive attitude towards life; with the assistance of family members and social service units, they can travel between home and their workplace without fear. In addition, improving social support from friends and enhancing the religious participation of the elderly with visual impairment can reduce loneliness and depressive symptoms [92]. The above information shows the high incidence rate of loneliness among middle-aged and elderly people with visual impairment. During the COVID-19 prevention period, social support measures to reduce such loneliness should be given high priority to ensure that middle-aged and elderly people with visual impairment can continue to live alone, and social support seems to be an important influencing factor.

## Figures and Tables

**Table 1:** Demographic and socioeconomic characteristics (*n* = 456).

Variable	*n* (%)
Gender	
Male	272 (59.6)
Female	184 (40.4)
Average age	54.1 (7.8) years
Educational level	
Junior and below	122 (26.8)
Senior high	196 (42.9)
College and university	94 (20.6)
Graduate school	44 (9.6)
Marital status	
Single	154 (33.8)
Married	240 (52.6)
Others (divorced)	62 (13.6)
Types of job	
White collar	20 (4.4)
Blue collar	254 (55.7)
Others	22 (4.8)
Unemployed	160 (35.0)
Monthly salary	
USD 525 and below	182 (37.9)
USD 526–1050	135 (29.6)
USD 1051~1580	83 (18.2)
USD 158~2100	36 (7.9)
USD 2100 and above	20 (4.4)
Average salary	USD 737.2
Religion	
Buddhism	96 (21.1)
Taoism	128 (28.1)
Christianity	42 (9.2)
Catholic	10 (2.2)
Other religions	28 (6.1)
None	152 (33.3)
Living Style	
Living alone	72 (15.8)
Living with family	350 (76.8)
Living with friends and family	16 (3.5)
Other	18 (3.9)
Ability to move independently	
Able to act independently	302 (66.2)
Not able to act independently	154 (33.8)
Ability to go out alone (*n* = 302)	
Using the cane	249 (82.5)
Using residual vision	44 (14.6)
Using guide dogs	9 (2.9)
Causes of Blindness	
Disease	250 (54.8)
Congenital blindness	154 (33.8)
Other (accidental injury)	52 (11.4)
Important support and companionship in life	
Parents	158 (34.64)
Spouse	146 (32.01)
Children	65 (14.25)
Siblings	64 (14.03)
Relatives and friends	14 (0.30)
Volunteers	9 (0.01)

**Table 2:** Differences in scores on the loneliness scale (*n* = 456).

Loneliness Classification	Minimum Value	Maximum Value	*n*	(%)
Low level	22	34	90	19.7%
Indicated a moderate level	36	47	224	49.1%
Indicated a moderately high level	52	64	134	29.4%
Indicated a high level	65	70	8	1.8%

**Table 3:** The loneliness level of the participants by different sociodemographic characteristics (*n* = 456).

Variable	Groups	*n*	Mean ± SD 44.05 ± 8.62	*p*-Value	Scheffe’s Post Hoc
Gender	Male	272	43.15 ± 9.72	T = 1.539	
	Female	184	44.47 ± 9.55		
Age (years)	45–50	190	43.99 ± 9.02	F = 2.424	
	51–60	160	45.98 ± 8.83		
	61–70	106	41.24 ± 8.26		
Educational level	Junior and below	122	43.48 ± 8.39	F = 1.834	
	Senior high	196	44.62 ± 9.36		
	College and university	94	46.05 ± 6.74		
	Graduate school	44	45.05 ± 8.62		
Marital status	Single	154	44.30 ± 9.00	F = 2.394	
	Married	240	43.77 ± 8.63		
	Others (divorced)	62	45.19 ± 6.74		
Have a job or not	Yes	296	43.20 ± 8.35	T = −3.211 ***	2 > 1
	No	160	47.16 ± 8.76		
Types of job	White collar	20	44.00 ± 7.19	F = 1.118	
	Blue collar	254	43.95 ± 8.47		
	Others	22	43.36 ± 8.68		
	Unemployed	160	47.76 ± 8.76		
Monthly salary	USD 525 and below	182	45.21 ± 8.84	F = 11.344 ***	4 > 3, 4 > 2, 4 > 1
	USD 526–1050	135	44.96 ± 9.32	*p* = 0.000	
	USD 1051~1580	83	43.56 ± 8.84		
	USD 1580~2100 (and above)	56	39.20 ± 5.50		
Religious	Yes	304	43.97 ± 7.16	T = −3.204 ***	
	No	152	48.68 ± 7.31	*p* = 0.000	
Religion	Buddhism	96	41.27 ± 7.34	F = 1.594	
	Taoism	128	44.14 ± 7.59		
	Christianity	42	40.76 ± 9.64		
	Catholic	10	45.40 ± 7.29		
	Other religions	28	45.20 ± 11.76		
Lifestyle	Living alone	72	47.40 ± 7.38	F = 10.528 ***	1 > 2, 1 > 3
	Living with family	350	40.88 ± 8.80	*p* = 0.000	
	Living with friends and family	16	42.87 ± 4.30		
	Others	18	41.00 ± 7.83		
Ability to move independently	Yes	302	42.64 ± 8.48	T = −5.042 ***	1 > 2
	No	154	47.83 ± 8.25	*p* = 0.000	
Cause of visual impairment	Disease	250	43.56 ± 9.78	F = 1.901	
	Congenital blindness	154	45.29 ± 11.00	*p* = 0.151	
	Other (accidental injury)	52	42.88 ± 10.14		

*** *p* < 0.001.

**Table 4:** Analysis of differences in social support scores (*n* = 456).

Four Dimensions of Social Support	Minimum Value	Maximum Value	Mean ± SD	Ranking
Emotional or appraisal support	4.00	16.00	9.56 ± 2.89	3
Instrumental or tangible support	4.00	18.00	9.20 ± 3.52	4
Companionship or belonging support	4.00	39.00	9.74 ± 3.54	2
Self-esteem support	4.00	16.00	9.96 ± 2.94	1
Total score and mean ± SD	16.00	63.00	38.63 ± 9.95	

**Table 5:** The social support of the participants by different sociodemographic characteristics (*n* = 456).

Variable	Groups	*n*	Mean ± SD 38.63 ± 9.95	*p*-Value	Scheffe’s Post hoc
Gender	Male	272	37.86 ± 9.39	T = −1.358	
	Female	184	39.15 ± 10.30		
Age (years)	45–50	190	38.32 ± 9.71	F = 2.191	
	51–60	160	39.86 ± 10.93	*p* = 0.113	
	61–70	106	37.35 ± 8.20		
Educational level	Junior and below	122	35.96 ± 10.06	F = 4.519 **	4 > 1
	Senior high	196	38.45 ± 9.86	*p* = 0.004	
	College and university	94	39.43 ± 9.69		
	Graduate school	44	42.98 ± 10.01		
Marital status	Single	154	36.97 ± 10.18	F = 7.697 **	2 > 1
	Married	240	40.08 ± 10.05	*p* = 0.001	
	Others (divorced)	62	39.18 ± 7.45		
Have a job or not	Yes	296	39.90 ± 9.32	T = −3.360 ***	1 > 2
	No	160	37.04 ± 8.88	*p* = 0.000	
Types of job	White collar	20	39.60 ± 6.49	F = 2.504	
	Blue collar	254	37.94 ± 10.09	*p* = 0.054	
	Others	22	38.36 ± 10.81		
	Unemployed	160	37.74 ± 8.88		
Monthly salary	USD 525 and below	182	36.66 ± 9.41	F = 6.865 *	4 > 1
	USD 526–1050	135	39.55 ± 9.90	*p* = 0.023	
	USD 1051~1580	83	38.94 ± 10.07		
	USD 1580~2100 (and above)	56	41.27 ± 9.70		
Religious	Yes	304	39.25 ± 7.16	T = 1.586	
	No	152	37.75 ± 7.31		
Religion	Buddhism	96	39.33 ± 10.91	F = 1.856	
	Taoism	128	39.69 ± 8.27	*p* = 0.118	
	Christianity	42	42.00 ± 11.24		
	Catholic	10	34.20 ± 11.45		
	Other religions	28	37.42 ± 7.95		
Lifestyle	Living alone	72	35.47 ± 9.47	F = 7.719 ***	2 > 1, 2 > 4
	Living with family	350	39.34 ± 10.07	*p* = 0.000	
	Living with friends and family	16	37.00 ± 4.06		
	Other (placement)	18	35.00 ± 7.09		
Ability to move independently	Yes	302	39.44 ± 10.85	T = 3.116 *	1 > 2
	No	154	37.16 ± 7.75	*p* = 0.035	
Cause of visual impairment	Disease	250	38.75 ± 10.03	F = 1.850	
	Congenital blindness	154	37.73 ± 9.40	*p* = 0.159	
	Other (accidental injury)	52	40.76 ± 10.98		

* *p* < 0.05, ** *p* < 0.01, *** *p* < 0.001.

**Table 6:** Analysis of the relationship between loneliness and social support.

	Loneliness	1	2	3
Social support	−0.572 ***			
Emotional or appraisal support	−0.471 ***			
Instrumental or tangible support	−0.556 ***	0.752 ***		
Companionship or belonging support	−0.521 ***	0.726 ***	0.736 ***	
Self-esteem	−0.608 ***	0.628 ***	0.484 ***	0.529 ***

1. Emotional or appraisal support; 2. Instrumental or tangible support; 3. Companionship or belonging support. *** *p* < 0.001.

**Table 7:** Stepwise multiple regression analysis of social support on loneliness.

Order of Variables Entered	R	R Square R2	R Square Change	F Change	Sig. F Change	Standardized Coefficients Beta
Self-esteem	0.342	0.117	0.117	40.118	0.000	−0.227
Instrumental or tangible support	0.410	0.168	0.050	30.326	0.001	−0.204
Companionship or belonging support	0.457	0.198	0.040	26.385	0.001	−0.193

## Data Availability

The data can be accessed upon reasonable request to the authors.
